# The Expression of CD30 Based on Immunohistochemistry Predicts Inferior Outcome in Patients with Diffuse Large B-Cell Lymphoma

**DOI:** 10.1371/journal.pone.0126615

**Published:** 2015-05-14

**Authors:** Xiaoxiao Hao, Xiaolei Wei, Fen Huang, Yongqiang Wei, Hong Zeng, Linwei Xu, Qinjun Zhou, Ru Feng

**Affiliations:** Department of Hematology, Nanfang Hospital, Southern Medical University, Guangzhou, China; University of Nebraska Medical Center, UNITED STATES

## Abstract

The prognostic value of CD30 expression indiffuse large B-cell lymphoma (DLBCL)remains controversial. Herein, we performed this retrospective study to investigate the clinical and prognostic significance of CD30 expression in patients with DLBCL.Among all the 146 patients, the expression of CD30 was observed in 23 cases (15.7%).The DLBCL patients with CD30 expression showed more likely to present B symptoms, bone marrow involvement, non-germinal centre B-cell-like (Non-GCB) DLBCL, BCL-2 and Ki-67overexpression(p<0.05). Patients with CD30 expression showed significantly poor overall and event-free survivalcompared with CD30 negative patients(p = 0.031 and 0.041, respectively), especially those with the high intermediate/high-risk international prognostic index (IPI)(p = 0.001 and 0.007, respectively). The prognostic value of CD30expression retained in DLBCL patients treated with eitherCHOP (cyclophosphamide, doxorubicin, vincristine,prednisone) or R-CHOP(rituximab+CHOP). The multivariate analysisrevealed that the expression of CD30 remained an unfavorable factor for both overall and event-free survival (p = 0.001 and 0.002, respectively).In conclusion, these data suggest that CD30 is expressed predominantly in Non-GCBDLBCL. The expression of CD30 implied poor outcomein DLBCL patientstreated with either CHOP or R-CHOP, especially those with the high intermediate/high-risk IPI, possibly indicating that anti-CD30 monoclonal antibody could be of clinical interest.

## Introduction

Diffuse large B-cell lymphoma (DLBCL), characterized by a high degree of heterogeneity in immunophenotype, pathogenetics, and clinical response, is the most common type of non-Hodgkin lymphoma(NHL)[1].The introduction of rituximab in immunochemotherapy has dramatically improved the outcome of patients with DLBCL [2–4]. Still, approximately 40% of patients with DLBCL suffer relapse and eventually die due to the disease [5], which highlights the need to construct prognostic models that can guide risk-justified treatment selection. International prognostic index (IPI) remains a valuable tool for risk stratification of DLBCL patients in the rituximab era [6, 7]. However it does not identify individual patients who will suffer a particularly aggressive clinical course, given that these patients can be found in the same subgroup. These prognostic variables are considered to be proxies for the underlying cellular and molecular variation within DLBCL.

CD30, a 120-kd transmembrane cytokine receptor of the tumor necrosis factor receptor (TNFR) family, is an important immune marker for the diagnosis of classical Hodgkin Lymphoma and anaplastic large cell lymphoma and carry a favorable prognosis[8, 9].Recent results indicate that CD30 expressionhad high prognostic relevance to the clinical outcome of DLBCL patients treated with the R-CHOP chemotherapy regimen [10, 11].However, the prognostic value of CD30 expression in DLBCL has been controversial and itstill remains unknown whether the prognostic value of CD30 expression can be applied to all the therapeutic regimens and, most importantly, if it can improve the prognostic profile based on the IPI. Therefore we performed this study to explore theprognostic value of CD30 expression in DLBCL patients with different treatment and whether CD30 expression has an independent prognostic value when compared with the IPIat diagnosis.

## Patients and Methods

### Patient population

All 146 patients consecutively diagnosed as de novo DLBCL with the available CD30 expression statusinNanfang Hospital between January, 2006and February, 2013 were further confirmed according to WHO classification. Patients were excluded if they were HIV-positive, or had various other types of DLBCL, including primary mediastinal, central nervous system, intravascular and testicular lymphomas, transformed NHL and posttransplant lymphoproliferative disorder. All patients were treated with CHOP (cyclophosphamide, doxorubicin, vincristine, and prednisone) or R-CHOP (rituximab plus cyclophosphamide, doxorubicin, vincristine, and prednisone).This study was approved by the Ethics Committee of Southern Medical University affiliated Nanfang Hospital. All patients had provided written informed consent themselves or their guardians prior to treatment allowing the use of their medical records for medical research.

### Immunohistochemistry (IHC)

The specimens from formalin-fixed and paraffin-embedded samplesat the time of initial diagnosis were collected for histological review and immunohistochemical analysis. IHC was carried out using a peroxidase-conjugated labeled dextran polymer method as our previously described[12]. Rabbit monoclonal antibody for CD30 (clone EP154, 1:50 dilution) was from ZSGB-BIO, Beijing, China. The other markers assessed in the present study included CD10, BCL-6, MUM-1, BCL-2 and Ki-67(ZSGB-BIO, Beijing). EBV was detected bysitu hybridization technique using a fluorescein-conjugated EBER oligonucleotide probe (Leica, America).A total of 200 cells in 5 well-preserved areas were scored for overall staining intensity and the percentage of the positively stained cells. All the slides were reviewed blindly by two experienced pathologists, with discrepant cases being jointly reviewed by a multihead microscope.CD30 and EBV staining in more than 20% of the malignant cells were considered positive, as previously described [10, 11, 13]. The cases were considered positive if 30% or more of the tumor cells were stained with CD10, BCL6, MUM1 and BCL-2. Ki67 staining in more than 85% of the malignant cells was considered overexpression as previous study [14]. Germinal center B-cell-like (GCB) and non-germinal center B-cell-like (Non-GCB) DLBCL were classified according to the algorithm described by Hans et al [15].

### Statistical analysis

Distributions of variables between the different groups were carried out by Mann-Whitney.Overall survival (OS) and event-free survival (EFS) were analyzed by Kaplan-Meier method and compared by the log-rank test. OS was definedfrom the time of diagnosis to the date of any cause to death or last follow-up[16]. EFS was defined from the time of diagnosis to the date of relapse,progression,death or last follow-up[16].Univariate and multivariate analyses were assessed by Cox proportional hazard regression model.Factors found to be significant in the univariate analysis were included in the multivariate analysis.All*p* values were two-sided and the significance was defined as *p*<0.05. Data were analyzed by the Statistical Package for Social Sciences 13.0.

## Results

### Patients’ characteristics

Of these 146 patients included, fifty-two patients were women and the ratio of male-to-female was 1.81:1. The median age was 49 years old (ranged 15 to 82 years), which is similar to three other recent studies of Chinese DLBCL patients [17–19], but much younger than those reported for DLBCL populations in the Western countries [20, 21]. Thirty-four patients (23.3%) were >60 years and 90 patients (61.6%) were in advanced stage (stages III and IV). Fifty-five patients had B symptoms and Eighty-eight (59.3%) had an elevated lactate dehydrogenase (LDH). Based on the IPI, 80 patients (55.0%) were in the low/low intermediate group.Sixty-two patients were treated with CHOP and others treated with R-CHOP.Baseline clinical features at the time of diagnosis are listed in **Table 1.**

**Table 1:** Clinical characteristics of patients according to CD30 expression.

		CD30		
Characteristics	Total	Negative	Positive	*P*-value
Age				0.849
≤60y	112(76.7%)	94(76.4%)	18(78.3%)	
>60y	34(23.3%)	29(23.6%)	5(21.7%)	
Gender				0.573
Female	52(35.6%)	45(36.6%)	7(20.4%)	
Male	94(64.4%)	78(63.4%)	16(69.6%)	
Performance status				0.489
0–1	104(71.4%)	89(72.4%)	15(65.2%)	
2–4	42(28.6%)	34(27.6%)	8(34.8%)	
B symptoms				0.001
No	85(60.7%)	79(66.4%)	6(28.6%)	
Yes	55(39.3%)	40(33.6%)	15(71.4%)	
Extranodal sites				0.608
0–1	77(52.7%)	66(53.7%)	11(47.8%)	
≥2	69(47.3%)	57(46.3%)	12(52.2%)	
Ann Arbor stage				0.396
Ⅰ/Ⅱ	56(38.43%)	49(39.8%)	7(30.4%)	
Ⅲ/Ⅳ	90(61.6%)	74(60.2%)	16(69.6%)	
LDH				0.389
Normal	58(40.7%)	47(38.2%)	11(47.8%)	
Elevated	88(59.3%)	76(61.8%)	12(52.2%)	
BM involvement				<0.001
No	120(82.2%)	107(87.0%)	13(56.5%)	
Yes	26(17.8%)	16(13.0%)	10(43.5%)	
IPI				0.466
0–2	80(55.0%)	69(56.1%)	11(47.8%)	
3–5	66(45.0%)	54(43.9%)	12(52.2%)	
COO				0.020
GCB	50(34.2%)	47(38.2%)	3 (13.0%)	
Non-GCB	96(65.6%	76(61.8%)	20(80%)	
BCL-2				0.041
negative	42(36.5%)	39(40.6%)	3(15.8%)	
positive	73(63.5%)	57(59.4%)	16(84.2%)	
Ki-67				0.048
Negative	34(26.4%)	30(27.8%)	4(19.0%)	
Positive	95(73.6%)	78(72.2%)	17(81.0%)	

LDH, Lactate dehydrogenase; BM, bone marrow;IPI,internationalprognosticindex

COO, cell of origin;GCB,germinal center B-cell like.

### The expression and prognosis of CD30 in DLBCL

We reviewed CD30 expression in a total of 146 de novo DLBCL cases and found 15.7% (23/146)of DLBCL patients were positive**(S1 Fig)**. BCL-2 and Ki-67 overexpression were detected in 63.5%and 73.6% of all patients. EBV was detected in 4.1% (6/146)of all patients.CD30 positive cases had a higher incidence of B symptoms (*p* = 0.001), bone marrow involvement (*p*<0.001),BCL-2 and Ki-67 overexpression (*p* = 0.041 and 0.048, respectively).The other clinical characteristics including age (*p* = 0.849), gender (*p* = 0.573), LDH (*p* = 0.389), performance status (*p* = 0.489), Ann Arbor stage (*p* = 0.396), extranodal sites (*p* = 0.608) and IPI (*p* = 0.466) showed no significant differences in DLBCL patients with and without the expression of CD30.According to the Hans’ algorithm the GCB DLBCL was applied to 50 of 146 cases (34.2%), the other 96 were of the Non-GCB DLBCLs. CD30 is expressed predominantly in Non-GCB DLBCL (*p* = 0.020).

**Fig 1:**
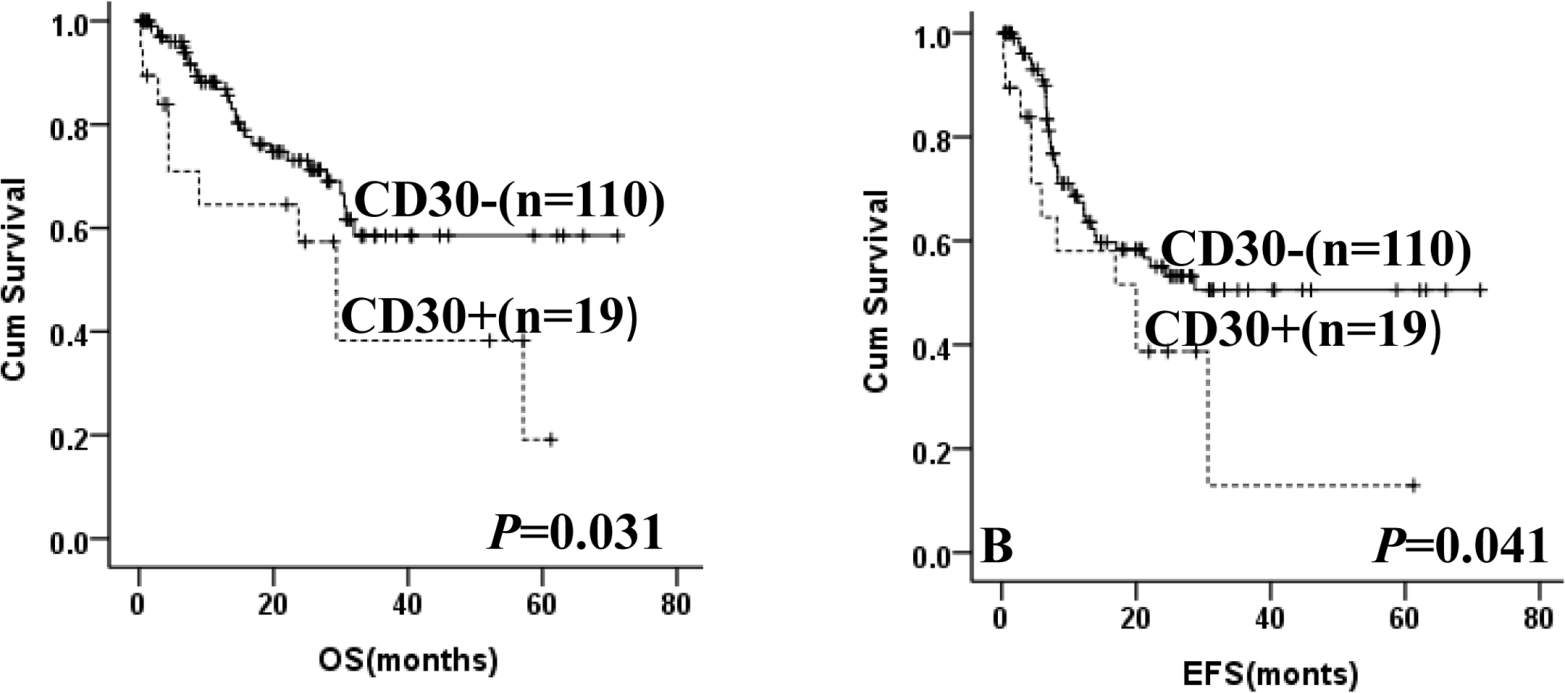
Kaplan-Meier curve of overall survival (A) and event-free survival (B) in DLBCL patients according to CD30 expression.

**Fig 2:**
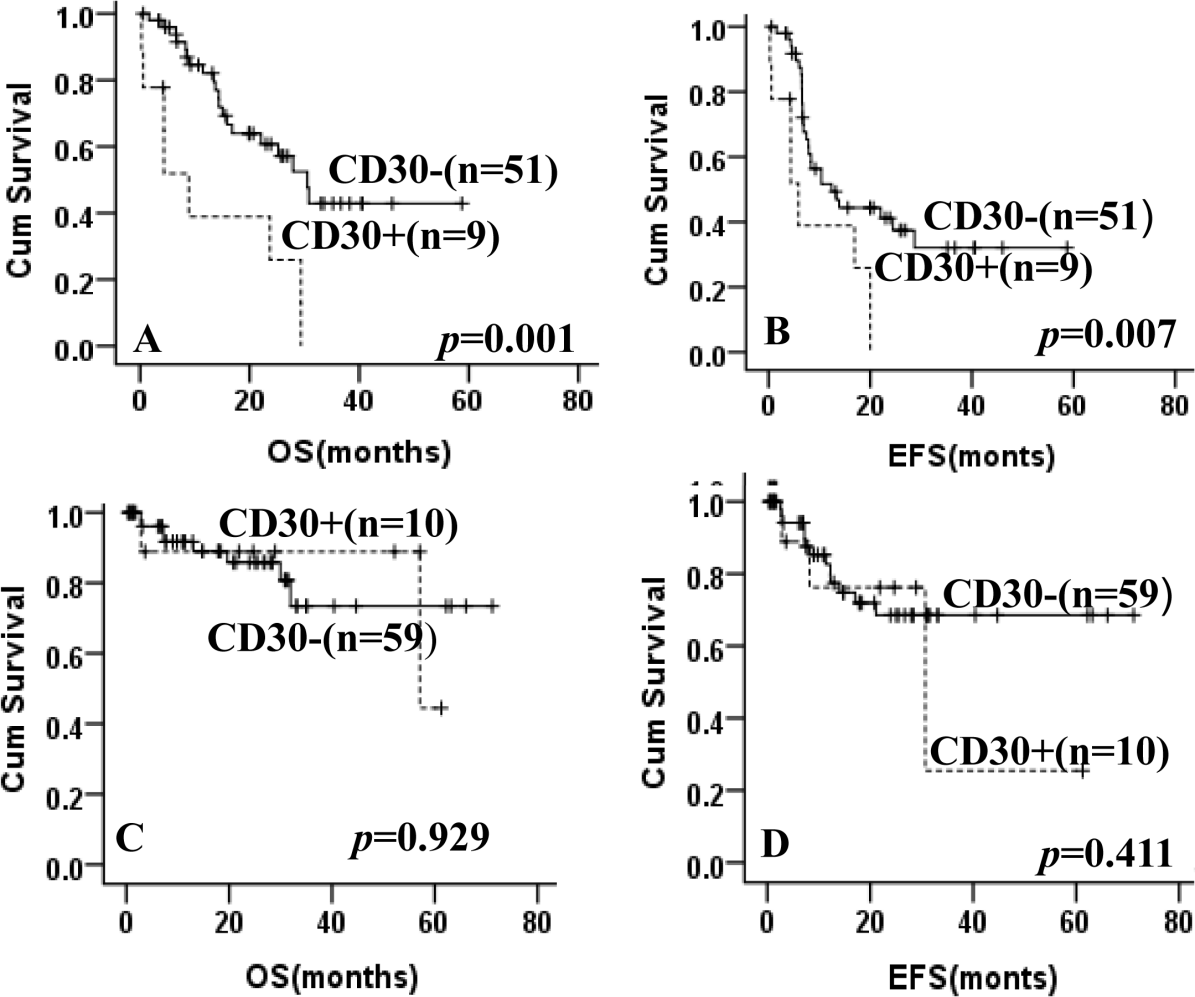
Kaplan-Meier curve for overall survival (OS) and event-free survival (EFS) according to the expression of CD30 and IPI. OS (A) and EFS (B) for high intermediate/high IPI risk patients (IPI = 3–5) with and without CD30 expression; OS (C) and EFS (D) for low/ low intermediate risk IPIpatients (IPI = 0–2)with and without CD30 expression.

**Fig 3:**
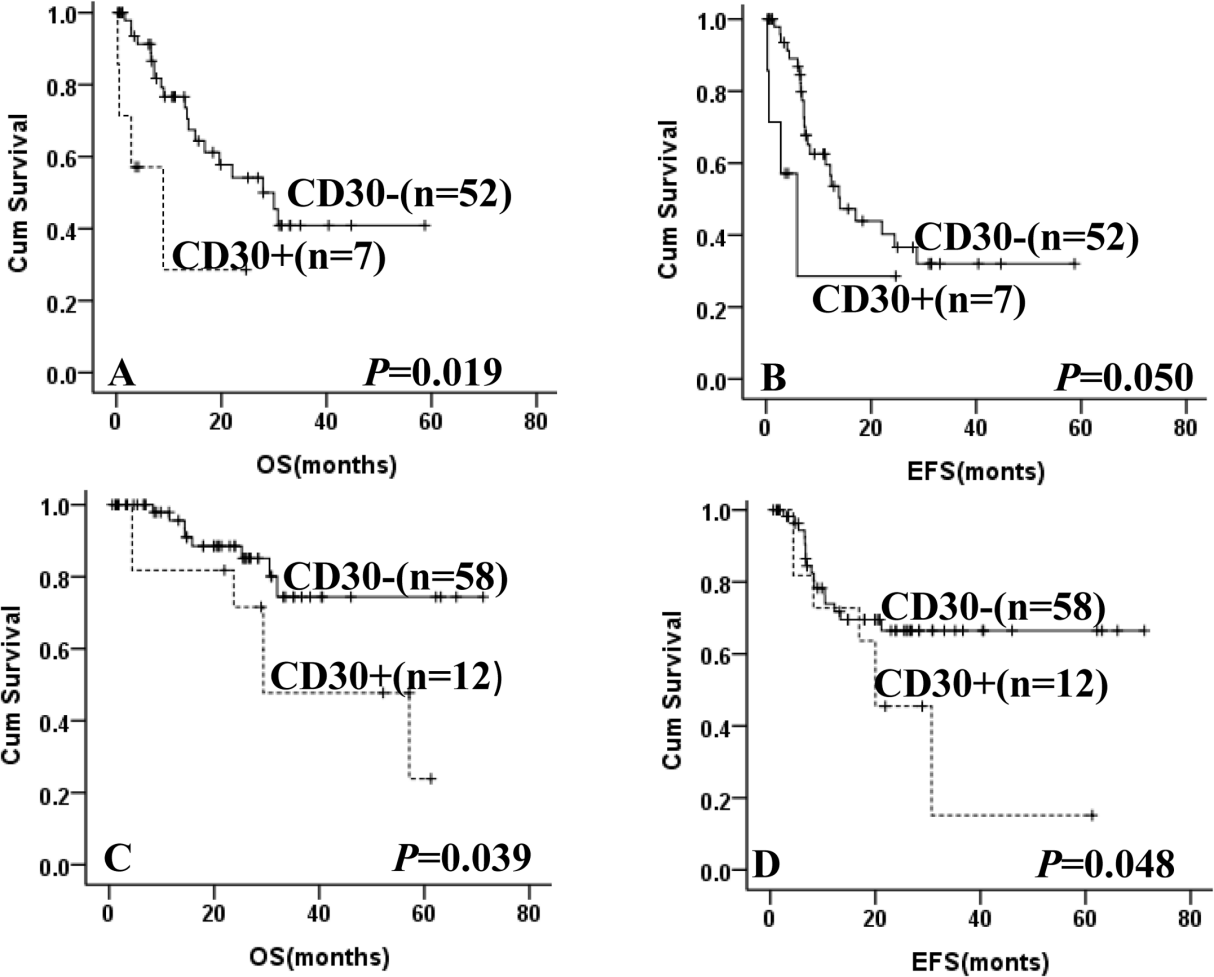
Kaplan-Meier curve for overall survival (OS) and event-free survival (EFS) according to the expression of CD30 and treatment. OS (**A**) and EFS (**B**) according to CD30 expression in DLBCL patients treated with CHOP. OS (**C**) and EFS (**D**) according to CD30 expression in DLBCL patients treated with R-CHOP.

After EBV-positive cases were excluded, patients with CD30 expression showedsignificantinferior OS and EFS compared with CD30-negative patients (**Fig 1**). The 5-year OS was 19.1% in patients with CD30 expression versus 58.5% for patients without CD30 expression (*p* = 0.031). The 5-year EFS was 12.9% for CD30-positive patients versus 58.4% for CD30-negative patients (*p* = 0.041).Furthermore, in the high intermediate/high group, patients with CD30 expressionimplied a poor OS and EFS (not reached versus 42.9% of 5-year OS, *p* = 0.001; not reached versus 32.1% of 5-year EFS, *p* = 0.007, **Fig 2A and 2B**), while no significant difference was observed in the low/ low intermediate group (44.5% versus 73.4% of 5-year OS, *p* = 0.929; 25.4% versus 68.5% of 5-year EFS, *p* = 0.411, **Fig 2C and 2D**).All patients were divided into CHOP and R-CHOP group according to treatment.In the patients treated with CHOP, CD30 expression also showed a trend to predict poor for OS and EFS (not reached versus 40.9% of 5-year OS, *p* = 0.019; not reached versus 32.0% of 5-year EFS, *p* = 0.050, **Fig 3A and 3B**).In the R-CHOP group, CD30 expression was still associated with a shorter OS and EFS as compared with CD30-negative patients (23.9% versus 74.4% of 5-year OS, *p* = 0.039; 15.2% versus 66.5% of 5-year EFS, *p* = 0.048, **Fig 3C and 3D**).

Multivariate analysis including all the significant factors in the univariate analysis showed that CD30 expression independent of LDH and B symptoms was aninferior predictor for OS (HR = 4.710; 95% CI = 1.964–11.295, *p* = 0.001)and EFS(HR = 3.393; 95% CI = 1.560–7.380,*p* = 0.002). The multivariate survival analysis was shown in **Table 2**.

**Table 2:** Multivariate Cox regression analysis for survival.

Prognostic factors	HR	95%CI	*P*-value
Overall survival			
CD30 positive	4.710	1.964–11.295	0.001
LDH elevated	5.842	1.939–17.603	0.002
B Symptoms	2.292	1.044–5.034	0.039
Performance status 3–4	0.813	0.368–1.795	0.608
Extranodal sites ≥2	1.306	0.421–4.047	0.644
IPI 3–5	1.561	0.405–6.022	0.518
Event-free survival			
CD30 positive	3.393	1.560–7.380	0.002
LDH elevated	3.431	1.469–8.016	0.004
B Symptoms	1.665	0.898–3.086	0.106
Bone marrow involvement	1.472	0.798–2.716	0.216
Extranodal sites ≥2	1.201	0.459–3.143	0.709
Ann Stage III~IV	1.628	0.614–4.317	0.328
IPI 3–5	1.177	0.434–3.198	0.749

LDH, Lactate dehydrogenase; IPI,internationalprognosticindex; HR, hazard ratio

95%CI, 95confidence interval

## Discussion

In the present study, weevaluated the clinical impact of CD30 expression in a cohortofpatients with de novo DLBCL. The expression of CD30 was associated with a shorter OS and EFS in DLBCL patients treated with either CHOP or R-CHOP. Multivariate analysis revealed that the expression of CD30 retained anindependent predictive factor associated with poor OS and EFS in patients with DLBCL. However, CD30 only added statistically significant prognostic information in the high intermediate/high-risk IPI patients, but not in the low/low intermediate-risk IPI group.

Due to factors including difficulties in the standardization of the IHC staining method and evaluation of the results, there has been a wide range of reported incidence of CD30 (9.6% to 21%) among DLBCL samples[10, 11, 22].In our study, approximately 15.7% of the DLBCL patients were classified as CD30-positive using 20% as a cut-off value in our study. The CD30-positive patients showed a greater tendency toward the presence ofB symptoms, bone marrow involvement and Ki-67 overexpression. Previous study hasshowed that CD30 expression on normal cells is restricted to activated T and B lymphocytes[23–25] as well as the activated B cell-like (ABC) DLBCL has more frequent and higher CD30 mRNA expression compared with GCBDLBCL[26].DLBCL has been divided into germinal center B cell–like (GCB) and activated B cell–like (ABC) subtypes by gene expression profiling with different clinical outcome[26]. Due to a small panel of IHC stains not completely capturing information obtained from GFP and the difference in the immunostaining methodologies and evaluation of the results, the study on IHC-based algorithms predicting GEP analysis are controversial[27]. Despite these limitations, Hans’ algorithm with a high correlation with GEP results has been widely used in clinical practice[15]. In our study, 50 of 146 cases were categorized into the GC subtype and the other 96 were the non-GC subtype based on Hans’ algorithm.Itis also interesting to note thatCD30 expression was predominant in Non-GCB DLBCLdescribed by Hans’ algorithm and correlated with the expression of bcl-2 protein in this study, which may contribute to explain the poor outcome.When CD30 expression, Non-GCB DLBCL and bcl-2 overexpression were included in the multivariate analysis by Cox modeling, CD30 expression didn’t retain its prognostic value (Data was not shown). So CD30 expression may be an useful treatment target in Non-GCB DLBCLpatientsdistinguishing from GCB DLBCL.

The CD30 protein belongs to a large family ofthe TNFR superfamily[23]. The pleiotropic effects of CD30 signaling on tumor cells varies from enhanced proliferation and survival to induction of growth inhibition and cell death, mainly through activation of the nuclear translocation of members of the NF- κB transcription factor family and mitogen activated protein kinase (MAPK) pathways[28]. The various reports on CD30 expression as a prognostic marker in DLBCL treated with R-CHOP have beencontradictory[10, 11, 29]. Furthermore, the introduction of rituximab to chemotherapy has remarkably improved the survival and altered the predictive valuesof known prognostic factors in DLBCL[30, 31]. Our datasuggested that CD30 expression was associated with poor survival in DLBCL patients treated with either CHOP or R-CHOP. The further results of subgroup analysis combing CD30and IPI showed that CD30 expression could identify a subgroup of DLBCL patients with inferior clinical outcome from high intermediate/high-risk IPI subgroup.A recent study fromCollieet al.[11]showed thatCD30 expression is a predictive factor of poor survival in DLBCL patients treated with R-CHOP, in agreement with our finding.However, another study in a large cohort of de novo DLBCL patients revealed that CD30 expression could identify a superior clinical outcome subgroup of DLBCL patients[10].Variable results among studies may result from different antibodies, IHC staining/scoring method, sample sizes and the heterogeneity of patients. In the present report there has a high proportion (76.7%) of patients who were ≤60 years old and the expression of CD30 in patients ≤60 years old implied a poor survival, but not in patients>60 years (**S2 Fig**), which may cause the difference between the studies.

However, it should be noted that this study was a retrospective analysis based on a relatively small numbers of patients. The choice of patients might have been biased, and other unrecognized bias might have influenced the results. Therefore, these findings need to be confirmed by future prospective study of larger cohorts.To minimize the inherent biases of the study, we selected only patients with de novo DLBCL treated with standard first-line chemotherapy and excluded other presentations of DLBCL such as HIV-positive, primary mediastinal, central nervous system, intravascular and testicular lymphomas, transformed NHL andposttransplant lymphoproliferative disorder.

In summary, we have studied the expression of CD30 in an independent cohort of de novo DLBCL and found that CD30 expression was predominant in Non-GCBDLBCL. The expression of CD30 detected by IHCseems to be associated with an inferior clinical outcome in DLBCL patients treated with either CHOP or R-CHOP, especially those with the high intermediate/high-risk IPI, who may benefit from experimental therapies such as anti-CD30 monoclonal antibody.

## Supporting Information

S1 Fig Representative images of CD30 expression in patients with DLBCL. (A) CD30 staining in a negative case. (B) CD30 staining in a positive case (×400).
(TIF)

S2 Fig Kaplan-Meier curve for overall survival (OS) and event-free survival (EFS) according to the expression of CD30 and age. OS (**A**) and EFS (**B**) according to CD30 expression in DLBCL patients ≤60 years. OS (**C**) and EFS (**D**) according to CD30 expression in DLBCL patients >60 years.
(TIF)
