# Supplementary figures and images for: The Expression of CD30 Based on Immunohistochemistry Predicts Inferior Outcome in Patients with Diffuse Large B-Cell Lymphoma
Source: PLoS One. 2015 May 14;10(5):e0126615. doi: 10.1371/journal.pone.0126615 (PMC4431801; doi:10.1371/journal.pone.0126615)

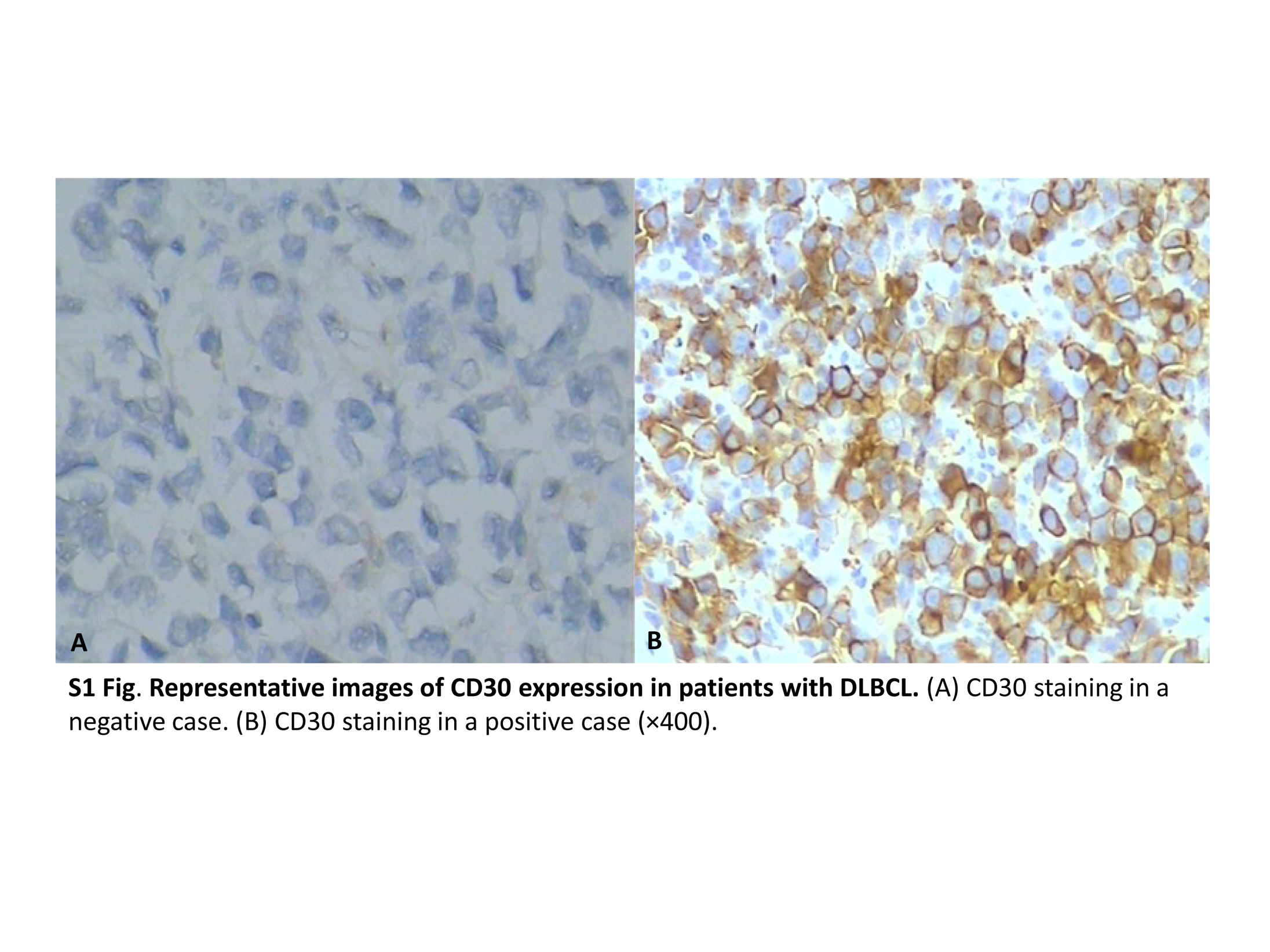

Supplement: S1 Fig — (A) CD30 staining in a negative case. (B) CD30 staining in a positive case (×400). (TIF) [file pone.0126615.s001.tif]

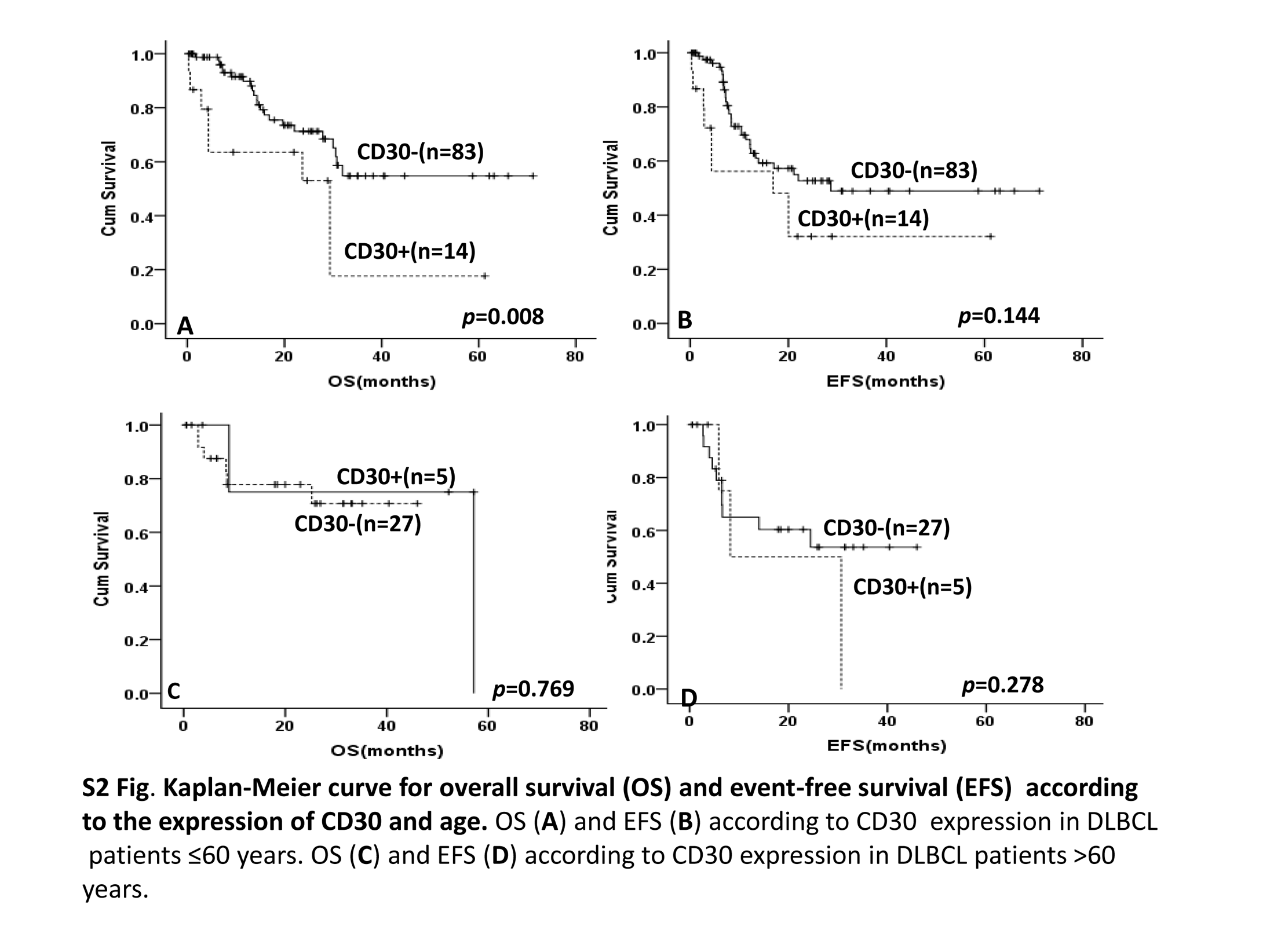

Supplement: S2 Fig — OS (A) and EFS (B) according to CD30 expression in DLBCL patients ≤60 years. OS (C) and EFS (D) according to CD30 expression in DLBCL patients >60 years. (TIF) [file pone.0126615.s002.tif]
